# Assessment of Multiple Sclerosis Disability Progression Using a Wearable Biosensor: A Pilot Study

**DOI:** 10.3390/jcm10061160

**Published:** 2021-03-10

**Authors:** Gianmarco Abbadessa, Luigi Lavorgna, Giuseppina Miele, Alfredo Mignone, Elisabetta Signoriello, Giacomo Lus, Marinella Clerico, Maddalena Sparaco, Simona Bonavita

**Affiliations:** 1 Department of Advanced Medical and Surgical Sciences, University of Campania “Luigi Vanvitelli”, 80138 Naples, Italy; gianmarcoabbadessa@live.com (G.A.); luigi.lavorgna@policliniconapoli.it (L.L.); giusy.mielee@gmail.com (G.M.); alfredservice.international@gmail.com (A.M.); elisabetta.signoriello@unicampania.it (E.S.); giacomo.lus@unicampania.it (G.L.); ms.86@hotmail.it (M.S.); 2 University of Turin, 10126 Torino, Italy; marinella.clerico@unito.it

**Keywords:** disability assessment, digital health, accelerometer

## Abstract

Background: The evaluation of walking activity of people with multiple sclerosis (pwMS) is desirable. We evaluate the power of the correlation of motor parameters detected by the accelerometer in the Samsung Gear S2 smartwatch with multiple sclerosis (MS) disability measures and patient reported outcomes (PROs). Methods: We enrolled 25 relapsing remitting MS patients. We assessed disability with the expanded disability status scale, two-minute walking test (2MWT), timed 25-foot walk test (T25FWT), and nine-hole peg test. We collected PROs measuring fatigue, ambulatory ability, depression, quality of life, and bladder/bowel function. Participants were asked to wear the accelerometer for a period of 30 days. Results: The Spearman’s rank correlation coefficient showed a moderate negative correlation between the patient-determined disease steps (PDDS) score with the mean steps/day, a strong negative correlation between the PDDS score with the maximum number of daily steps (MNDS) and a moderate negative correlation between the fatigue severity scale score and MNDS. A moderate negative correlation between MNDS and the 2MWT and a moderate negative correlation between MNDS and the T25FW was found. Conclusion: Our results suggest that motor parameters derived from the accelerometer could be a reliable measure of motor disability in pwMS.

## 1. Introduction

The disability assessment of people with multiple sclerosis (pwMS) is based on the evaluation of walking ability [1]. The most commonly used MS disability metrics [2] are the expanded disability status scale (EDSS) and the ambulation index (AIx); however, in outpatient clinical practice, these metrics fail to intercept minimal changes in walking performance. Standard clinical performance-based measures, such as the two-minute walking test (2MWT) and timed 25-foot walk test (T25FWT), provide objective pictures of walking ability in a clinic-based setting but may not reflect ambulatory skills in the real-world environment [3]. Patient-reported outcomes (PROs) of ambulatory function, i.e., patient-determined disease steps (PDDS), are limited by variability in the self-perception of walking ability [4].

The ability to monitor disease progression in pwMS and catch walking changes is crucial for therapy adjustments [2]. To overcome these limitations, objective evaluation of the walking activity of pwMS in daily life is desirable [5,6] and remote monitoring with wearable devices may be useful for documenting patient status [7,8].

Comfortable and non-invasive devices could be useful to monitor pwMS remotely and intercept disability progression [4,9]. Several studies have shown that the steps/day parameter is a reliable measure of free-living walking behavior in pwMS [6]. Previous studies in MS using commercial research-grade accelerometers (e.g., ActiGraph) demonstrate moderate to strong correlations between step and activity counts and standard MS disability measures [4]. We aimed to evaluate the power of the correlation of motor parameters detected by the accelerometer installed in the Samsung Gear S2 smartwatch with standard MS disability measures and PROs.

## 2. Experimental Section

In this cross-sectional interventional study with a medical device, we consecutively enrolled 25 subjects with MS-RR from the Multiple Sclerosis Center of the University of Campania Luigi Vanvitelli, after receiving and signing informed consent. The inclusion criteria included: a clinically definite diagnosis of the RR form of MS, 18 to 65 years of age, EDSS > 3 and < 6, relapse-free and steroid-free in the last three months, able to walk for at least two minutes with or without aid, access to WiFi Internet at home or patients’ community, and a willingness to continuously wear a device for a month. Exclusion criteria included: major musculoskeletal, cardiovascular, and/or respiratory comorbidities that could substantially impair physical activity and/or confound results; clinical relapse within three months of study entry; and a mental functional system > 1 at EDSS (to exclude patients with cognitive impairment that might interfere with compliance in the use of the device). We assessed, at study entry, the patients’ disability using the following measures: maximum walking distance (MWD), EDSS, 2MWT, T25FWT, and the nine-hole peg test (9HPT). 

The MWD in routine clinical practice is reported by the patients. In this study, we assessed the MWD by observing patients walking unassisted along a twenty-meter aisle, without rest, until the onset of symptoms.

Moreover, the following PROs were collected: the nine-item fatigue severity scale (FSS) (to assess fatigue severity and its effects on daily living) [10], the Beck depression inventory-II (BDI-II) [11], the nine-item patient health questionnaire (PHQ9) [12] (to evaluate the presence of depression), the patient-reported outcome indices for multiple sclerosis (PRIMUS) (a 15-item assessment to evaluate changes in activities of daily living) [13], the short form 36 (SF-36) (to investigate health-related quality of life) [14], the Italian version of the PDDS [15] (to evaluate perceived disability), the bladder control scale (BLCS) [16] and the bowel control scale (BWCS) [16]. At study entry and during the following week, participants were provided with an accelerometer (Samsung Gear S2) and were trained on the set-up and use of the device. They were instructed to wear it as a wrist bracelet, and to charge the battery every two days. Participants were asked to wear the device for a 30 days period on their non-dominant wrist to avoid the detection bias due to wrist movements occurring during housework or talking with gesturing since the dominant hand is used for many activities that may result in erroneous steps [17]; they were also asked to wear the device as much as possible except while swimming and driving a car (to avoid erroneous steps being recorded) and were instructed to continue with their normal daily lives.

The outcome from the accelerometer was expressed as the total daily steps averaged over all valid days of the 30-day period (mean steps/day). Valid days were determined based on adequate wearing time; all participants with three or more valid days of data were included in the analyses; this is the minimum necessary for a reliable estimate of usual behavior [18]. For quality control, days in which < 300 steps were recorded were excluded from the analysis to minimize potential bias, such as non-wearing of the device that day. This threshold of 300 steps is based on observed ranges of the step count in previous studies in MS [4]. The Samsung Gear S uses an accelerometry-based algorithm during walking/running and predictive equations during cycling. Consistent with previous research [19], step count estimates for the Samsung Gear S are acceptable (within four to six percent of the reference). The data generated by the Samsung Gear S2 (daily step-count) were transferred to the smartphone through Bluetooth technology. Once on the smartphone, the biosensor data were displayed in the respective app of the sensor, and at the same time, were sent to secure cloud storage and on a secure sockets layer (SSL) encrypted website accessible to the physician. The mobile application was compatible with the Android system and the users could download it from Google Play. The Samsung Gear S2 is GDPR compliant.

### Statistical Methods

Continuous variables were expressed as the mean ± standard deviation (SD) or median with range. The prevalence of categorical variables was expressed as a number and percentage. Fisher’s exact test and Chi-square test was used to estimate the frequency of demographic and clinical characteristics expressed as categorical variables of cases. The motor parameters of the accelerometer taken into consideration and treated as continuous variables were:

Mean steps/day (see above).

Median daily steps: the median of the daily steps taking into account all valid days in a period of 30 days.

Minimum number of daily steps: minimum number of steps performed in a day, taking into account all valid days in a period of 30 days.

Maximum number of daily steps: maximum number of steps performed in a day, taking into account all valid days in a period of 30 days.

As in previous works [20], the EDSS was treated as a binary variable, with a cut-off of 4. In fact, up to the EDSS score of 4, walking autonomy minimally affects the final score. Therefore, two groups were identified based on EDSS: mild/moderate disability (EDSS ≤ 4) and severe disability (EDSS > 4). Even the PROs, as in previous works, were treated as binary variables (cut-off: median value) [20]. Therefore, for each scale, two groups were identified: presence or absence of fatigue according to the FSS scale (cut-off: 4.33), presence or absence of depression according to the PHQ-9 scale (cut-off: 6), presence or absence of depression according to the BDI-II scale (cut-off: 12), presence or absence of walking difficulties according to the PDDS scale (cut-off: 2.5). A Student’s t-test was used to evaluate the groups’ (mild/moderate disability vs. severe disability; absence of fatigue vs. presence of fatigue; absence of depression vs. presence of depression; absence of walking difficulties vs. presence of walking difficulties) differences for the mean accelerometer data (mean steps/day, median steps, the minimum number of daily steps, the maximum number of daily steps) by comparing the identified groups. Finally, the Spearman’s rank correlation coefficient (SRCC) was applied to evaluate the correlation between the accelerometer data (mean steps/day, median daily steps, the maximum and the minimum number of daily steps) and the demographic and clinical characteristics of patients. These differences were considered statistically significant for *p* values < 0.05.

## 3. Results

We collected data on 25 patients. The demographic and clinical characteristics and accelerometer parameters of pwMS are shown in Table 1. Despite a lower mean steps/day (4960 steps) in the group of patients with severe disability (EDSS > 4) compared to the mean steps/day (5545 steps) of patients with mild/moderate disability (EDSS < 4), this difference was not statistically significant (*p* = 0.28). There was a statistically significant difference between pwMS not reporting walking difficulties (PDDS score < 2.5) compared to those reporting walking difficulties (PDDS score > 2.5) in the mean steps/day (*p* = 0.03), in the minimum daily steps (*p* = 0.03) and the maximum number of daily steps (*p* = 0.0005) (Table 2). There was also a statistically significant difference (*p* = 0.04) in the maximum number of daily steps between PwMS who did not report fatigue (FSS score < 4.33) and those who reported fatigue (FSS score > 4.33) (Table 2).

The SRCC showed a moderate negative correlation between the score at the PDDS scale with the mean steps/day (r^2^ = −0.4; *p* = 0.05), a strong negative correlation between the score at the PDDS with the maximum number of daily steps (r^2^ = −0.650; *p* = 0.001) and a moderate negative correlation between the score at the FSS scale and the maximum number of daily steps (r^2^ = −0.473; *p* = 0.04). A moderate correlation between the maximum number of daily steps and the 2MW (r^2^ = −0.429; *p* = 0.04) and a moderate negative correlation between the maximum number of daily steps and the T25FW (r^2^ = 0,4; *p* = 0,05) was found. No correlation was revealed between EDSS, MWD, 9HPT, BLCS, BWCS, BDI-II, PHQ9 and PRIMUS and the accelerometer measures. Table 3 reports in full the SRCC analysis between the maximum daily steps and the most relevant clinical and demographic variables.

## 4. Discussion

The mean steps/day count in our cohort (5239 steps per day) is consistent with previous studies in MS [9]. Our results showed a moderate to strong negative correlation between the PDDS score and the mean steps/day and the maximum number of daily steps and a moderate negative correlation between the FSS score and the maximum number of daily steps. These results are in line with previous works exploring the correlation between the mean steps/day and PROs scores. A study by Block and colleagues [9] investigated the correlation between different PROs and the mean steps/day, revealing the strongest correlations with walking performance and fatigue scales, suggesting a reasonable influence of fatigue on the pwMS walking endurance in the real-world environment. The correlation with the scales assessing the presence of depression, pain, or bowel/bladder incontinence was much smaller. Similarly, our results disclose little or no correlation between the bladder/bowel disturbance or depression scales and the parameters detected by the accelerometer. We might expect that depression could influence walking behavior (i.e., reducing the urge to go out). However, the lack of correlation between depression and the wearable biosensor parameters in our sample might be explained by the rather low scores achieved by pwMS at the BDI and PHQ-9 questionnaires. 

No correlation between EDSS and MWD and the parameters detected by the accelerometer was found, whereas a moderate negative correlation between the maximum number of daily steps and 2MWT and T25FWT was revealed. These data are not in line with the literature that showed a moderate to strong correlation between the mean steps/day and the clinical tests, even the EDSS [9,20]. However, MWD measured during the clinical examination is greatly influenced by the disease status, patients’ mood, and patients’ fatigue. Indeed, previous findings showed high day-to-day variability in the walking ability of pwMS [21,22]. Moreover, pwMS achieve their exhaustion limit when performing the maximum walking distance in the outpatient setting [21]. On the contrary, the mean steps/day is a measure detected in a real-life context, explaining the stronger correlation with PDDS rather than with the standard MS disability measures.

Among the motor parameters detected by the accelerometer, the maximum number of daily steps was shown to be the parameter that best correlates with the standard MS disability measures and the PROs. The mean steps/day has been widely confirmed, in previous studies, as the most accurate motor parameter to measure disability in pwMS. However, our results suggest that even the maximum value of steps could be considered a reliable parameter for this purpose. In only 30 days of evaluation, the presence of days in which few steps are taken for reasons other than disability (i.e., low adherence), could weigh enormously on the mean and median values, thus explaining the lack of correlation between the mean and median daily steps and the correlation (although weak) with PROs. Therefore, our data suggest that the maximum value of steps, not being influenced by patients’ adherence, could be a better parameter in evaluating ambulatory performance. Future studies to confirm this hypothesis are advisable.

We believe that adding the output of a wearable biosensor in the clinical follow-up of pwMS will ensure better monitoring. The information provided by clinical, laboratory, radiological, PROs and wearable biosensors data will furnish the MS specialist with a thorough picture of the patient’s clinical condition and help to guide therapy choices.

## 5. Limitations

One major limitation is the lack of a control group since the inclusion of healthy controls would provide a solid background for walking habits. Indeed, whilst the MS-specific metrics do not apply to putative healthy controls, the overall differences between pwMS and unaffected healthy individuals may provide important information regarding the degree of affection of the entire patient population.

## 6. Conclusions

In conclusion, in line with previous studies, our results suggest that motor parameters derived from an accelerometer could be a reliable measure of motor disability in pwMS. Moreover, given the better correlation with both the objective and subjective disability measures and the independence from adherence, we propose that recording the maximum number rather than the mean of daily steps might be preferrable. Longitudinal studies to assess the usefulness of the accelerometer output (combined with clinical, radiological and PROs measures), in detecting individual disability changes are desirable.

## Figures and Tables

**Table 1:** Demographic and clinical characteristics of PwMS.

Parameter	Value
Female sex (*n*,%)	12, 48
Age (mean ± SD)	40.08 ± 8.87
Disease duration in days (mean ± SD)	3406 ± 2416
EDSS (baseline) (mean ± ds)	4.45 ± 1.39
Years of education (mean ± ds)	14.23 ± 2.53
2MW (mean ± ds)	135 ± 43.71
MWD (mean ± ds)	289.95 ± 2
T25FW (mean ± ds)	5.83 ± 1.67
9HPT dominant hand (mean ± ds)	28.15 ± 9.37
9HPT non-dominant hand (mean ± ds)	30.60 ± 8.60
PRIMUS (mean ± ds)	6.75 ± 4.04
PHQ9 (mean ± ds)	6.62 ± 5.55
FSS (mean ± ds)	3.69 ± 14.98
BDI (mean ± ds)	11.16 ± 7.06
PDDS (mean ± ds)	2.20 ± 1.25
Mean steps/day (mean ± ds)	5239.16 ± 464.4577
Median daily steps (mean ± ds)	5140.2 ± 459.579
Minimum daily steps (mean ± ds)	1911.52 ± 327.096
Maximum daily steps (mean ± ds)	9019.44 ± 712.6276

9-HPT: nine-hole peg test, PRIMUS: patient-reported outcome indices for multiple sclerosis, EDSS: expanded disability status scale, FSS: fatigue severity scale, MaxSteps: maximum daily steps, MWD: maximum walking distance, PDDS: patient-determined disease steps, PHQ9: the nine-item patient health questionnaire, 2MW: two-minute walking test, T25FWT: timed 25-foot walk test, BDI-II: Beck depression inventory-II.

**Table 2:** Difference in the means of the motor parameters detected with the accelerometer in PwMS, divided into two categories for each test or clinical scale according to the defined cut-offs.

	Mean Steps/Day	*p* Value	Median Daily Steps	*p* Value	MinSteps	*p* Value	MaxSteps	*p* Value
EDSS ≤ 4	5545	0.28	5169	0.4	2359	0.17	9615	0.28
EDSS > 4	4960		4949		1678		8702	
BDI < 12	5415	0.20	5283	0.25	2182	0.051	9169	0.23
BDI > 12	4597		4587		1115		8136	
PHQ9 < 6	5289	0.41	5139	0.5	2022	0.25	8936	0.58
PHQ9 > 6	5037		5143		1467		9351	
FSS < 4.33	5855	0.16	5444	0.3	2564	0.06	10632	**0.04**
FSS > 4.33	4892		4968		1544		8112	
PDDS < 2.5	8169	**0.03**	5863	0.09	2566	**0.03**	11353	**0.0005**
PDDS > 2.5	4486		4610		1328		8112	

MinSteps: minimum daily steps, MaxSteps: maximum daily steps, EDSS: expanded disability status scale, PDDS: patient-determined disease steps, FSS: fatigue severity scale, BDI-II: Beck depression inventory-II, PHQ9: nine-item patient health questionnaire. Bold characters are statistically significant.

**Table 3:** Bivariate associations between the maximum value of steps and demographic and clinical variables, both objectively detected and reported by the patient. The heatmap (below) graphically depicts the direction of the correlation, with red tones trending towards a stronger association, negative or positive (± 1). Correlations are computed using the Spearman’s q.

	Sex	Age	EDSS	MWD	T25FW	PDDS	FSS	PHQ9	PRIMUS	2MW	MaxSteps
**Sex**	1										
**Age**	−0.302	1									
**EDSS**	−0.088	0.568	1								
**MWD**	0.0320	−0.458	−0.912	1							
**T25FW**	0.0557	0.665	0.466	−0.402	1						
**PDDS**	−0.198	0.608	0.398	−0.307	0.647	1					
**FSS**	−0.2944	0.306	0.187	−0.250	0.264	0.419	1				
**PHQ9**	−0.008	0.064	0.010	−0.228	−0.045	0.014	0.743	1			
**PRIMUS**	−0.208	0.027	0.167	−0.276	0.110	0.252	0.831	0.830	1		
**2MW**	0.213	−0.635	−0.535	0.578	−0.669	−0.459	−0.112	0.158	0.043	1	
**MaxSteps**	0.301	**−0.539**	−0.270	0.267	**−0.400**	**−0.650**	**−0.473**	−0.237	−0.357	**0.429**	1
*Heatmap*											
0–0.1 Absent		0.2–0.3 Low		0.4–0.5 Moderate		0.6–0.7 Strong		0.8–0.9 Very strong		1 Perfect	
0–(−0.1) Absent		(−0.2)–(−0.3) Low		(−0.4)–(−0.5) Moderate		(−0.6)–(−0.7) Strong		(−0.8)–(−0.9) Very strong		−1 Perfect	

PRIMUS: patient-reported outcome indices for multiple sclerosis, EDSS: expanded disability status scale, FSS: fatigue severity scale, MaxSteps: maximum daily steps, MWD: maximum walking distance, PDDS: patient-determined disease steps, PHQ9: nine-item patient health questionnaire, 2MW: two-minute walking test; T25FWT: timed 25-foot walk test. Bold characters are statistically significant.

## Data Availability

The data presented in this study are available on request from the corresponding author. The data are not publicly available due to ongoing longitudinal study.
